# Are Conventional Impressions Obsolete? A Narrative Review on the Applicability of Intraoral Scanners

**DOI:** 10.1111/adj.70015

**Published:** 2025-11-03

**Authors:** Ahmad Amro Baradee, Benedikt Christopher Spies

**Affiliations:** ^1^ Department of Prosthetic Dentistry, Center for Dental Medicine Medical Center ‐ University of Freiburg, Faculty of Medicine, University of Freiburg Freiburg Germany

**Keywords:** conventional impressions, digital impressions, digital workflow, intraoral scanner, scan

## Abstract

This narrative review examines the clinical indication, practical advantages and limitations of dental digital impressions as compared to conventional impression techniques. Digital impressions are scientifically approved for their high accuracy for single tooth restorations while enhancing workflow efficiency through seamless integration with computer‐aided design and computer‐aided manufacturing (CAD/CAM) systems. They improve both patient and clinician experiences by reducing discomfort, shortening treatment times and facilitating more direct communication with dental laboratories. Furthermore, digital records offer long‐term archiving benefits and enable direct comparisons with future scans, which can be valuable for monitoring treatment progress. Despite these advantages, digital impressions face challenges in specific clinical scenarios, including movable oral tissues, deep subgingival margins and long‐span prostheses. These limitations currently restrict their universal application across all dental indications. Nonetheless, digital impressions are increasingly being adopted in clinical practice across all dental specialties. As technological advancements of intraoral scanners continue, particularly improvements in scanning accuracy and the integration of artificial intelligence, the clinical utility of digital impressions is expected to expand further.

**Abbreviations**
AI: artificial intelligence
CAD: computer‐aided design
CAM: computer‐aided manufacturing
RPD: removable partial denture

**Summary**

- This review examines the capabilities and limitations of digital impressions, offering guidance for clinicians seeking to effectively integrate this technology into their practice.
- Digital impressions are rapidly evolving and challenging conventional methods across a broad range of applications, while introducing innovative digital solutions.
- With advances in scanner accuracy, digital impressions are set to become the norm, reducing material waste and optimising treatment protocols.

## 1 Introduction

Digital technology is transforming how dental impressions are taken, with intraoral scanners becoming increasingly common in clinical settings [1]. Intraoral scanners enable the acquisition of high‐resolution, three‐dimensional digital impressions and are increasingly regarded as a viable alternative to conventional impression materials [2, 3, 4]. Their adoption is driven not only by the broader shift toward digital workflows but also by demonstrated benefits in patient comfort, chairside efficiency and seamless integration with computer‐aided design and manufacturing systems [3, 4, 5, 6, 7].

Integrating digital impressions into clinical practice requires significant investment, both in financial resources and in training the clinical team [1]. Nonetheless, this investment is justified by the wide applicability of digital impression techniques across various indications, including single crowns, fixed dental prostheses, implant‐supported reconstructions, orthodontic appliances, occlusal splints and removable prosthodontics [2, 3, 4, 8, 9]. Consequently, numerous investigations have compared digital and conventional impression techniques, addressing parameters such as trueness, precision, clinical fit of restorations, procedure time and patient‐reported outcomes [2, 3, 5, 6, 8, 10, 11, 12]. Encouraging findings in favour of digital methods challenge the continued use of traditional impressions [2, 3, 4, 5, 6, 8, 9, 10, 11, 13].

This review aims to summarise current knowledge regarding these emerging approaches and provide an outlook on the future of dental impressions. To clarify their clinical applicability, this review organises findings according to the scanned region, focusing on the advantages and limitations of intraoral scanning techniques. Only studies published within the last decade are considered, as older scanning devices have largely become obsolete [2, 4, 14].

## 2 Clinical Application Areas

### 2.1 Short‐Span Scans of Natural Teeth

Digital impressions are most effective for single‐tooth restorations or short‐span fixed partial dentures [2, 10]. Studies have shown that restorations made from digital impressions provide a comparable fit to those created using conventional impressions [2]. Additionally, chairside restorations, which depend almost entirely on digital impressions, have added significant advantages to daily clinical care [4, 7]. However, dentists who intend to place indirect post‐and‐core restorations should consider digitising conventional impressions, as deep or complex canal preparations remain a challenge for intraoral scanners [15].

Similar to conventional triple tray impressions, digital impressions can be limited to the treated quadrant [16]. Scanning only one half of the jaw not only reduces clinical time but has also been shown to improve scan accuracy [17]. However, a study has found that bite registration tends to be more accurate when full‐arch scans are used [18].

### 2.2 Short‐Span Scans of Dental Implants

The results obtained with scan bodies for capturing the position of dental implants, for single‐tooth restorations or short‐span fixed partial dentures, are also considered clinically acceptable [11, 13]. However, dentists should be aware of factors that can influence the accuracy of scan bodies to ensure optimal outcomes [13, 19, 20]. To accurately capture peri‐implant soft tissues, a supplementary digital impression can be taken immediately after removing the healing abutment or provisional restoration [21]. This allows for clear visualisation of the soft tissue contours, which can guide the digital design of the final restoration. Alternatively, the provisional restoration can be scanned extraorally and then aligned with the digital impression to replicate its emergence profile in the final design. This indirect technique helps prevent inaccuracies caused by soft tissue collapse [21].

### 2.3 Full‐Arch Scans of Natural Teeth

One of the greatest challenges faced by intraoral scanners is capturing a full‐arch scan with high accuracy [4]. Because intraoral scanners capture only a limited area per image, full‐arch scans must be obtained by stitching together multiple images, a process that can introduce inaccuracies. This limitation becomes evident when comparing results from desktop scanners to those from intraoral scanners, as the accuracy of intraoral scanners tends to decrease with an increased scanning span [13].

Although full‐arch digital scans are currently less accurate than conventional methods [13], the discrepancy may be clinically acceptable depending on the intended application. For instance, full‐arch digital scans provide sufficient accuracy for primary models [22], implant surgical guides [2, 23], occlusal splints [9], fixed orthodontic retainers [24], analysis of orthodontic models [4] and aligner therapy [2]. This suitability extends even to challenging scenarios, such as orthodontic treatment with brackets, where digital impressions tend to be less problematic than conventional techniques [8].

**FIGURE 1:**
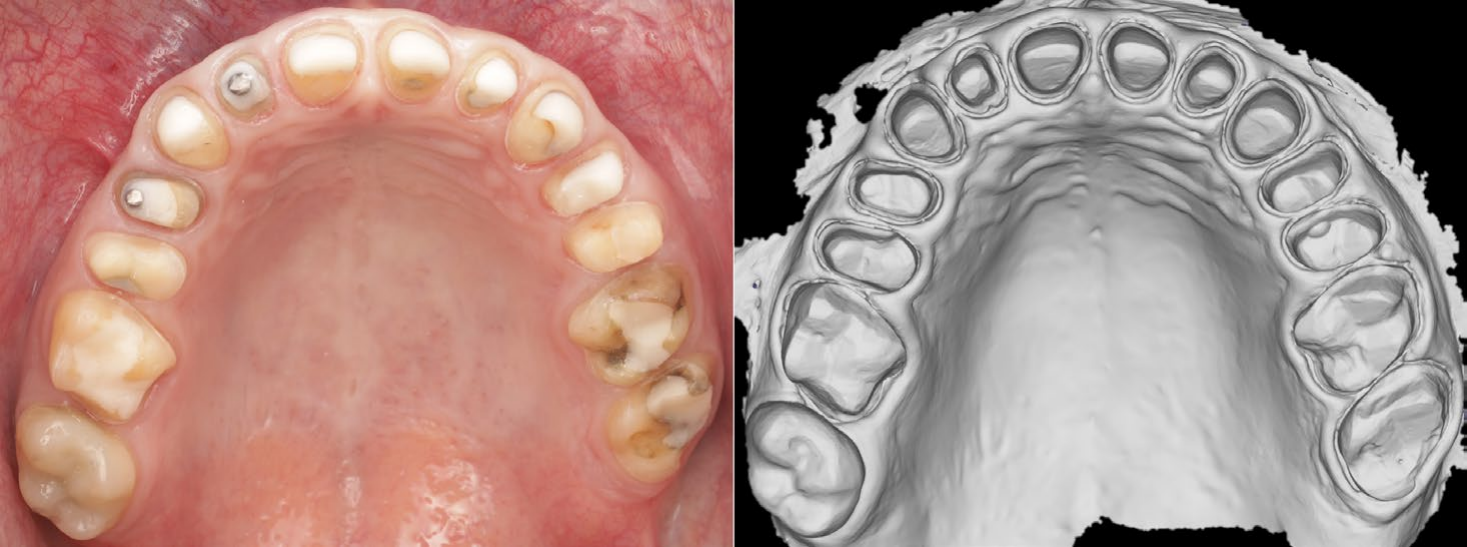
Digital impression of a full‐arch case. Intraoral scanning was successfully performed, offering significant advantages.

It can be assumed that the accuracy observed in short‐span restorations may also apply to full‐arch cases when the restorations consist of individual short‐span units rather than being splinted together. In such cases, inaccuracies resulting from the longer scan path are likely to slightly affect the occlusal contacts, which can be corrected through intraoral adjustments (Figure 1).

### 2.4 Full‐Arch Scans of Dental Implants

The inaccuracy of full‐arch scans is particularly discussed in the context of splinted implant‐supported full‐arch restorations [13, 19, 25, 26]. Such inaccuracies may result in an inadequate fit at the interface of screw‐retained restorations, potentially generating adverse mechanical stresses within the implant, restorative components and peri‐implant tissues [25, 27]. These stresses have been investigated in numerous in vitro studies and are often regarded as potential contributors to both biological and mechanical complications, including peri‐implant bone loss, screw loosening, screw fracture and structural failure of the implant or restoration [25, 27]. Current literature recommends achieving a passive fit for these restorations, as verified by the Sheffield Test, to prevent both biological and technical complications [13, 25, 27]. Additionally, many studies propose specific misfit thresholds (e.g., < 100 μm) as benchmarks for clinical acceptance [19, 27]. However, these thresholds have not been sufficiently investigated in clinical settings, and the clinical relevance of the reduced accuracy remains debatable [25, 27]. Notably, a recent systematic review found that full‐arch implant‐supported prostheses fabricated using intraoral scans achieve survival outcomes comparable to those produced using conventional impressions [19].

Nevertheless, various strategies have been explored to overcome the reduced accuracy of full arch scans. These strategies include the use of adjunctive components to improve the stitching of multiple scans [28, 29], modifying the scan protocol [30], superimposing a scan of a verification cast [31] and employing photogrammetry‐based techniques [28, 32]. One study has found photogrammetry to be even more accurate than conventional impression techniques for full‐arch implant prostheses, yet results across the literature remain inconsistent [32]. Recently, the Aoralscan Elite IPG (Shining 3D Tech Co.; Hangzhou, China) was introduced, integrating photogrammetry into an intraoral scanner, thereby eliminating the need for additional devices. This intraoral photogrammetry scanner seems to surpass the accuracy of regular intraoral scanners for splinted full‐arch restorations, although further studies are needed to confirm this finding [26, 28]. In parallel, smartphone‐based photogrammetry applications are emerging as a promising, more accessible alternative [33]. Dentists should carefully assess whether investing in photogrammetry is worthwhile, taking into account the number of full‐arch implant cases they manage.

### 2.5 Functional Areas

Scanning edentulous or partially edentulous jaws presents a challenge, especially in the mandibular region [2, 13, 29, 34], since intraoral scanners are unable to capture movable oral tissues during function. To address this limitation, various methods have been developed to digitise edentulous cases [35]. A straightforward approach begins with a primary digital impression, which serves as a viable alternative to conventional alginate impressions [22, 34]. From this, a custom tray is created using computer‐aided design and computer‐aided manufacturing (CAD/CAM). This custom tray captures a conventional functional impression and can also be designed to register jaw relations in the same visit. The resulting conventional functional impression is then digitised, enabling the digital design and fabrication of dentures [34, 35]. Following a digital process can reduce treatment time and the number of visits, which is particularly beneficial for elderly patients [35].

A novel hybrid approach combines a mucostatic digital impression of the edentulous arches with a digitised conventional mucocompressive impression, which captures the functional denture borders [34]. While its impact on denture retention and alveolar bone resorption remains unclear, this technique may offer advantages in cases that require a localised static impression, such as flabby ridges [34].

**FIGURE 2:**
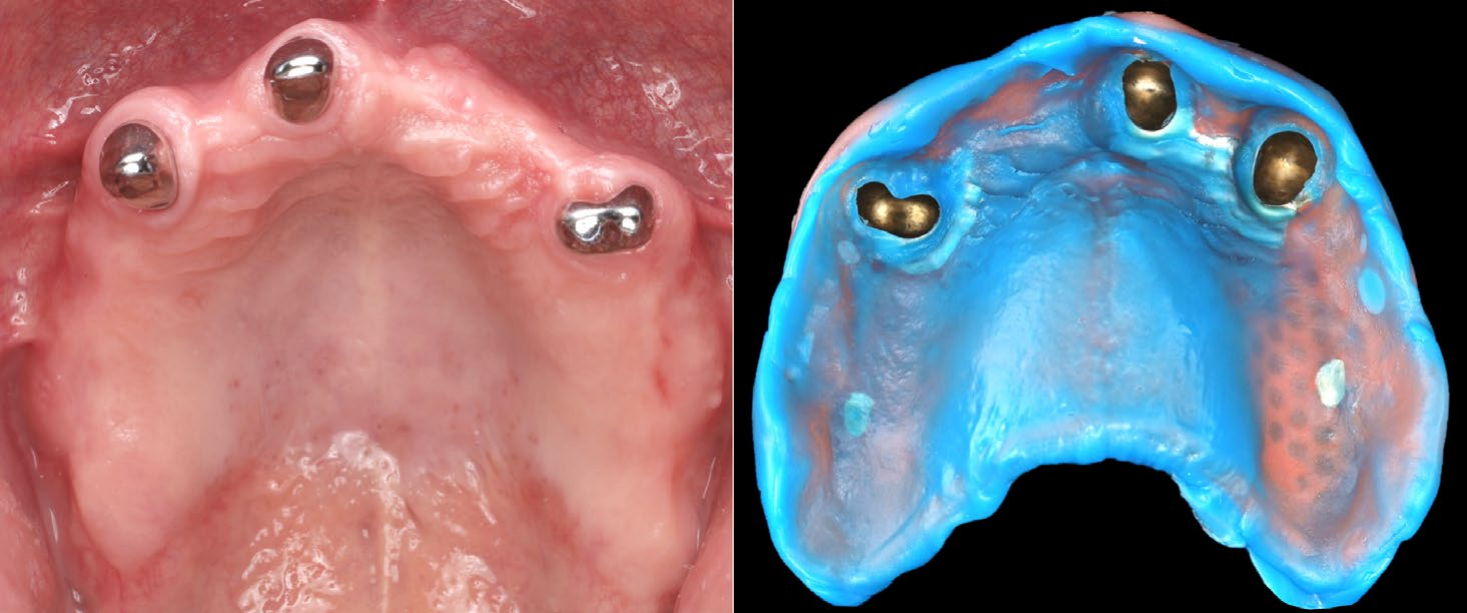
Relining impression of a telescopic prosthesis at the 1‐year follow‐up of a previously published case report [38]. When the telescopic crowns are properly seated, the intaglio surface of the prosthesis displaces the soft tissues. In such cases, conventional impressions remain the only viable method.

As with other procedures involving functional impressions, denture relining still primarily relies on conventional techniques (Figure 2). However, digitising a conventional relining impression along with the denture, and duplicating it digitally, offers an alternative to the conventional relining method [36]. Moreover, saving the digital design file of a denture enables fast reproduction when needed [36], which is especially beneficial in nursing homes, where dentures are often lost and access to dental care is limited [37]. For this reason, dentists are urged to proactively create digital scans of adequate existing dentures, a process that can be carried out using intraoral scanners.

**FIGURE 3:**
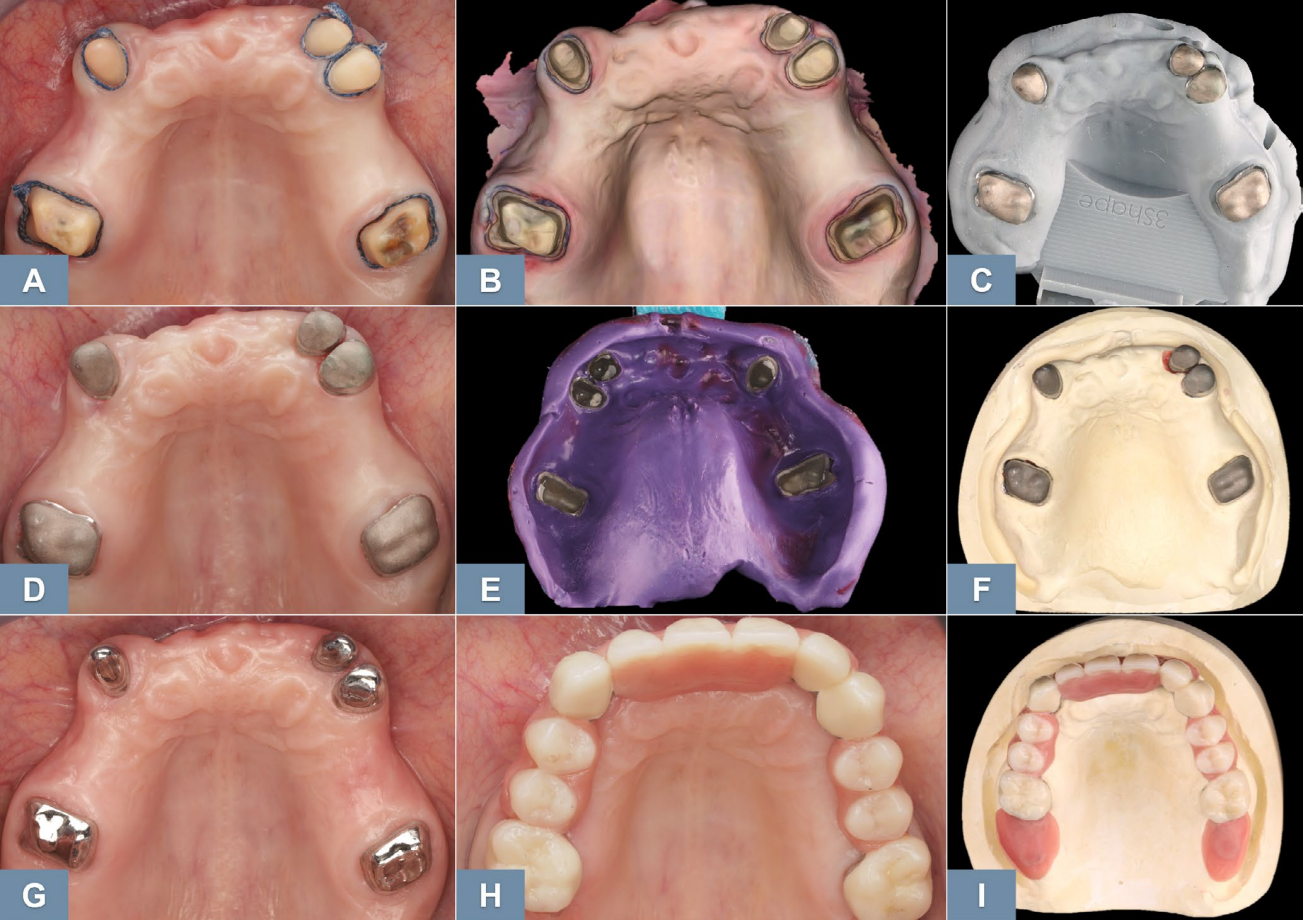
Optimised use of digital and conventional methods in a combined workflow for the fabrication of a telescopic prosthesis. (A) Gingival retraction. (B) Digital impression. (C) CAD/CAM telescopic crowns. (D) Clinical repositioning of the crowns. (E) Conventional pick‐up impression (polyether) using a CAD/CAM‐fabricated custom tray. (F) Working plaster model. (G) Cemented telescopic crowns. (H) Proper fit of the prosthesis. (I) Prosthesis placed on the working model.

Fabricating removable partial dentures (RPDs) based on intraoral scans has been shown to be feasible [39, 40]. However, the same challenges persist due to the inaccuracy of full‐arch scans and the inability to record functional movements of movable soft tissues [41]. For example, while the crowns supporting a removable prosthesis can be effectively fabricated from intraoral scan data, the prosthesis requires both a precise full‐arch fit at the teeth and accurate adaptation to the soft tissues at the prosthesis borders [39], both of which are still best achieved using conventional impressions (Figure 3). In vitro results indicate that although RPD frameworks fabricated from intraoral scans may exhibit reduced accuracy, the deviations may fall within clinically acceptable limits [40]. Furthermore, in vivo studies have reported favourable fit outcomes for RPD frameworks fabricated from intraoral scan data [42, 43]. Despite these promising results, long‐term clinical trials are still needed to assess the performance of scan‐based RPDs, beyond the initial fit of the frameworks. Notably, digitising only the laboratory steps in the fabrication process has demonstrated reliable clinical outcomes [44].

### 2.6 Bite Registration

Digital impressions also raise concerns about the accuracy of digitally acquired jaw relations which are typically recorded by scanning the buccal surfaces of the remaining teeth [13]. Recent in vitro studies suggest that the accuracy of digitally acquired jaw relations depends on the interocclusal space and the number of remaining teeth, with greater accuracy observed when there is a larger interocclusal space and a higher number of remaining teeth [45, 46]. However, in fully edentulous cases, where no teeth are present to guide this alignment, new instruments or techniques have been developed to capture jaw relations [35]. Once a digital jaw relationship has been registered, it can be subsequently reproduced to preserve both vertical and horizontal jaw relations. This presents a significant advantage and is typically achieved by aligning previous and current digital impressions.

Artificial intelligence (AI) has been proposed as a solution for predicting jaw relations (e.g., Bite‐Finder, Bite‐Finder AG; Basel, Switzerland). This approach is inherently a form of estimation and is only viable when sufficient occlusal surfaces are present [18, 45]. Nevertheless, such software could assist technicians in double‐checking the accuracy of digitally registered jaw relations.

Discarding conventional impressions also means moving away from traditional face‐bow registration. In response, digital face‐bow systems have been introduced [47, 48]. However, the practical advantages of a face‐bow remain a topic of debate [49, 50]. A recent study on short‐span restorations reported better results with a digital workflow that did not use a face‐bow registration, compared to a conventional workflow that included it [10]. Furthermore, many intraoral scanners now support dynamic occlusion scans, enabling the digital recording of actual mandibular movements [13, 51]. These patient‐specific mandibular motion recordings have been shown to outperform average‐value virtual articulators [51]. Alternatively, digital jaw tracking systems have been developed as specialised tools to capture mandibular movements and condylar position [52, 53].

**FIGURE 4:**
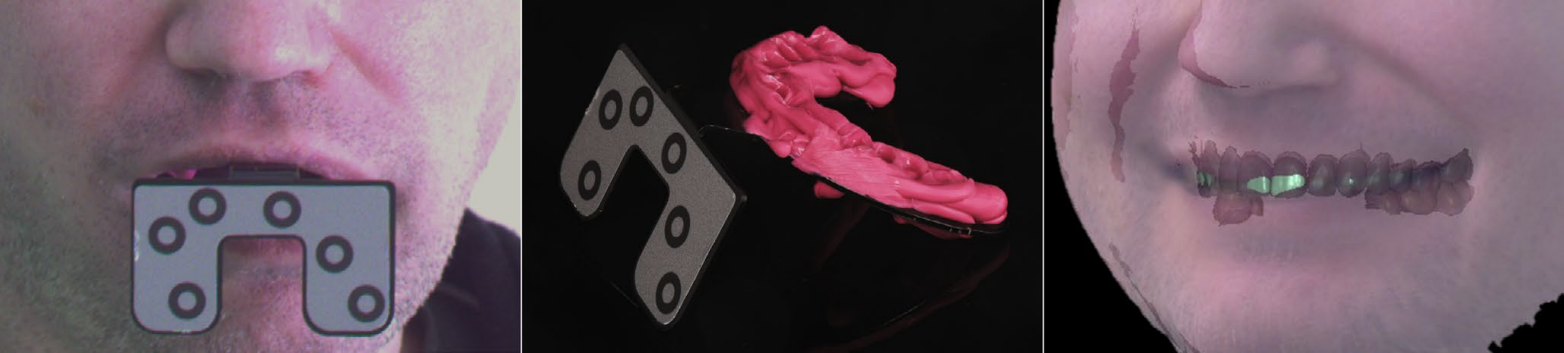
Transferring 3D facial scans using a transfer fork (Face Hunter, Zirkonzahn; Gais BZ, South Tyrol, Italy).

One indication for using a face‐bow, as reported in the literature, is when a change in vertical dimension is planned [50]. However, recording this change through clinical digital registration of the intended new jaw relation may provide accuracy that is comparable to, or even greater than, the traditional method of increasing the vertical dimension using the hinge movement of an articulator. Additionally, with the development of facial scanning technologies [47, 48], aesthetic evaluations for full‐arch restorations or for orthodontic treatments can be conducted more comprehensively. This digital approach offers a more detailed reference than relying solely on an occlusal plane orientation captured by a face‐bow (Figure 4).

### 2.7 Summary of Clinical Applications

The applications discussed above highlighted the increasing importance of intraoral scanners in contemporary dental practice. They already provide reliable results for short‐span restorations on both natural teeth and implants, offering accuracy comparable to conventional impressions while facilitating efficient chairside workflows. Although full‐arch scans exhibit slightly lower precision than conventional impressions, they remain sufficiently accurate for many clinical scenarios where minor deviations are not clinically significant. Challenges persist for implant‐supported full‐arch and long‐span restorations; however, ongoing advancements in scanning technology are steadily improving outcomes. Moreover, hybrid approaches that combine digital and conventional functional impressions effectively address the complexities of capturing dynamic soft tissue morphology. Beyond impressions, intraoral scanners facilitate digital bite registration, jaw motion tracking and integration with facial scanning.

## 3 Advantages and Capabilities

Matching the accuracy of conventional impressions alone may not be sufficient to justify a shift to digital impressions, particularly given the challenges and limitations of intraoral scanners. However, patient‐centered care is a core principle of modern dentistry, and numerous studies comparing patient experiences with digital and conventional impressions consistently show that patients find digital impressions more comfortable [2, 3, 5, 8, 10, 13]. For instance, challenges such as the difficulty of removing an impression tray due to improper blocking of undercuts or gagging during the impression process are avoided with digital impressions [2, 5]. Comfort is an important consideration, as future patients may increasingly expect this technology and actively choose clinics that offer the convenience of digital scanners. Additionally, patients are likely to favour dental practices that provide chairside same‐day restorations, which rely on digital impressions. These chairside restorations eliminate the need for temporary restorations, simplifying the treatment process by reducing both overall treatment time and the number of visits [7].

Not only do patients report a more positive experience with intraoral scanners, but dental professionals also note improvements in workflow and increased efficiency [3, 10, 11, 12]. Intraoral scanners are practical and accessible tools for practitioners of all skill levels, including dentists, dental students and dental assistants [11, 12]. However, experience with digital impressions does impact the total scanning time [11, 54] and may also affect the final accuracy [54]. Therefore, dentists should invest sufficient time mastering this new technique before giving an early negative judgement. Additionally, respecting factors such as ambient lighting [55] and adherence to a specific scanning protocol [30] can enhance the quality of the scan results [56].

Treatment time, along with patient comfort, is one of the most extensively studied factors when comparing digital and conventional impressions. Most studies suggest that digital impressions require less clinical time [2, 3, 6, 8, 19]. However, accurately measuring the duration of the impression procedure is complex, as several variables need to be considered [3] such as the type of intraoral scanner used, the hardware capabilities of the computing device, the time required for equipment preparation and the potential need for retaking an impression. Additionally, studies usually fail to account for all available conventional impression techniques, such as fast‐setting materials or triple tray impressions, which in some clinical cases could offer a time advantage. However, concerns like recovery time, as well as the impact of storage duration and temperature on dimensional stability, are completely eliminated with digital impressions [3]. Treatment time can also be reduced when final indirect restorations require minimal or no adjustments before placement. Evidence suggests that digital workflows reduce the need for adjustments [6, 10, 57]. However, one study found no significant difference [11].

Conventional impressions generate considerable material waste and demand physical transport to dental laboratories, having a negative impact on the environment. In contrast, digital impressions eliminate material consumption and can be transmitted instantly to technicians worldwide, significantly reducing the environmental footprint. Furthermore, digital impressions eliminate the need for disinfection solutions, reducing the use of disinfectants and minimising the risk of bacterial contamination for dental technicians [3]. They also eliminate the need for custom trays, impression adhesives and mixing tips, further reducing material consumption and waste. Additionally, reusing sterilised scan bodies has been shown to provide greater accuracy compared to reusing sterilised conventional impression posts, which are more prone to deformation during impression‐taking or model preparation [20]. However, further improvements are still needed to enhance the accuracy of sterilised scan bodies [20]. These material savings, along with reduced time for clinical professionals and technicians, may help explain the overall cost reduction reported for digitally manufactured single implant crowns [6], implant‐supported overdentures [58] and fixed orthodontic retainers [24].

**FIGURE 5:**
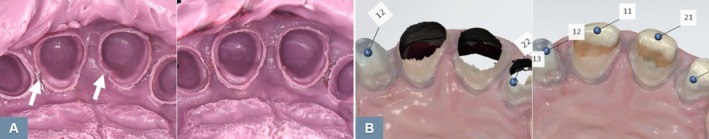
(A) Arrows indicate missing details in a polyether impression; these areas were targeted in a supplementary impression. (B) Efficient correction using the cut‐out‐rescan feature of a digital impression system.

Having a digital model available immediately after scanning offers significant advantages, as it allows the dentist to evaluate critical factors such as tooth reduction, undercuts and the path of insertion before sending the impression to the dental lab. If any adjustments are needed, the dentist can easily perform them and rescan the modified areas after selectively removing the previous data. However, these cut‐out‐rescan procedures may lead to less accurate outcomes, so dentists should aim to avoid them whenever possible [56, 59]. In contrast, when a conventional impression fails in certain areas, a supplementary impression is typically taken to focus on the problematic regions. This adds complexity to the laboratory workflow, extends chairside and laboratory time, increases material consumption and heightens patient discomfort (Figure 5).

Digital impressions also offer the advantage of passively capturing the oral condition, which is especially beneficial in cases of periodontal tooth mobility or dental trauma. This feature helps prevent the misplacement of teeth during the impression process. Additionally, scanners are particularly useful in cases of recession, where the interdental papillae are absent, an area where conventional impression materials often struggle [60]. However, deep undercuts between teeth can still pose challenges for intraoral scanners [54, 61].

**FIGURE 6:**
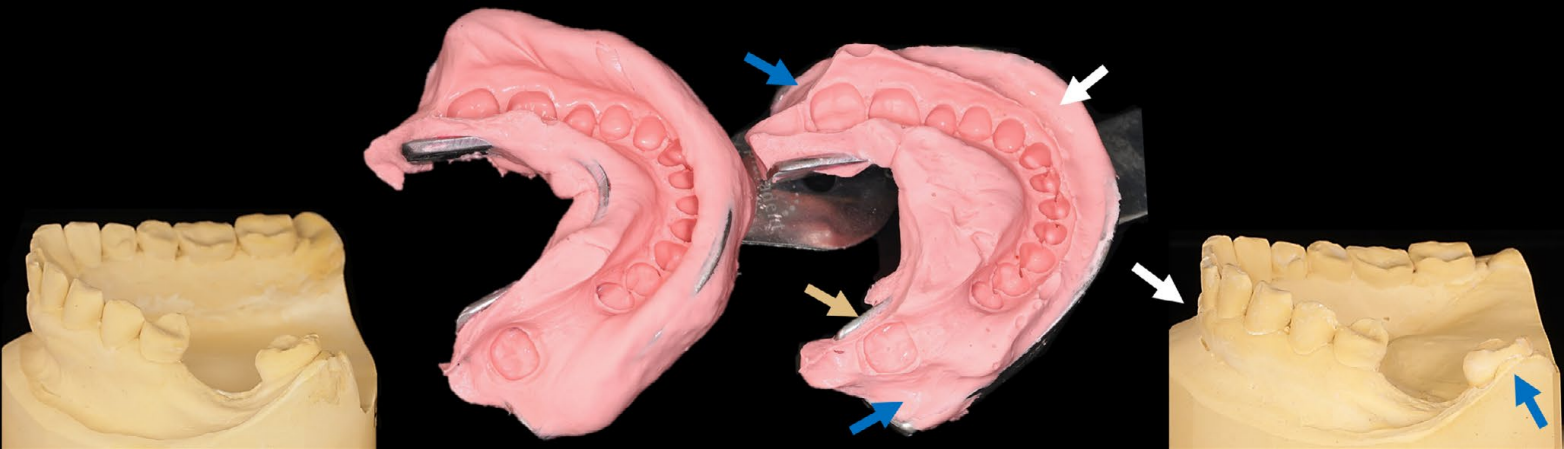
Adequate (left) and poor (right) alginate impressions with their corresponding plaster models. The poor outcome was due to insufficient retraction of the lip (white arrows), tongue (yellow arrow) and cheeks (blue arrows).

Managing nearby anatomical structures such as the tongue and cheeks is important to prevent them from being inadvertently scanned [56]. This issue is not exclusive to digital impressions, as conventional methods also face similar challenges, where retakes can be both costly and time‐consuming (Figure 6). However, digital impressions are increasingly supported by AI that can detect and correct inadvertent scanning of nearby structures or retractors [56].

In an increasingly aging society, intraoral scanners have the potential to enhance the treatment experience for elderly patients by offering greater comfort and ease [62]. Moreover, digital impressions can be taken in nursing homes, improving accessibility and promoting teledentistry for these individuals [4, 62]. Additionally, children tend to report a better experience and greater acceptance of intraoral scanners [5]. This is especially beneficial, as children may move or request sudden stops during a conventional impression process. Such interruptions are less disruptive during digital scanning, making it a more forgiving and flexible option for both younger and older patients.

Patients with limited mouth opening [39] or allergies to certain impression materials also benefit from the transition to digital scanners. Additionally, patients with oral defects, such as cleft palate or oroantral and oronasal communications may find digital impressions more comfortable and safer [63, 64] as conventional impressions in these cases can be not only uncomfortable but also potentially cause breathing difficulties.

With the obligation to confidentially archive dental records, storing conventional plaster models requires considerable space, which can be costly. Digitising these models offers a more efficient, space‐saving solution [2]. Digital impressions eliminate the need for an additional digitising step, as records are already in digital form. This simplifies archiving, allowing for efficient long‐term storage or the creation of duplicates for patients or other dental professionals. Furthermore, digital records are not subject to wear or degradation over time and can be superimposed with other digital impressions. This feature improves long‐term patient monitoring, aids in the early detection of changes, enhances treatment planning by enabling direct comparisons between past and present oral conditions, and could even be used for forensic purposes [4, 65]. To aid in this matching process, dentists are encouraged to always extend the scan area to include key anatomical landmarks, such as the hard palate, which can improve alignment accuracy between impressions [65].

## 4 Challenges and Limitations

Understanding common mistakes and limitations of intraoral scanners is essential to improve both the overall experience and treatment outcomes [13, 56, 66]. For example, intraoral scanners may face challenges with translucent or glossy surfaces [14, 67]. To address this, intraoral scanning powder can be used to improve the scanning results [66]. Moreover, in situations where translucent or glossy approximal surfaces are difficult to capture [67], technicians may compensate by slightly overestimating the approximal surfaces in the restoration design. This allows the dentist to make intraoral adjustments upon delivery to ensure an optimal fit.

Many intraoral scanners also include tooth shade detection capabilities [4], potentially supporting clinical decision‐making. Nevertheless, current evidence shows that the reliability of tooth shade determination varies across different scanners [68].

**FIGURE 7:**
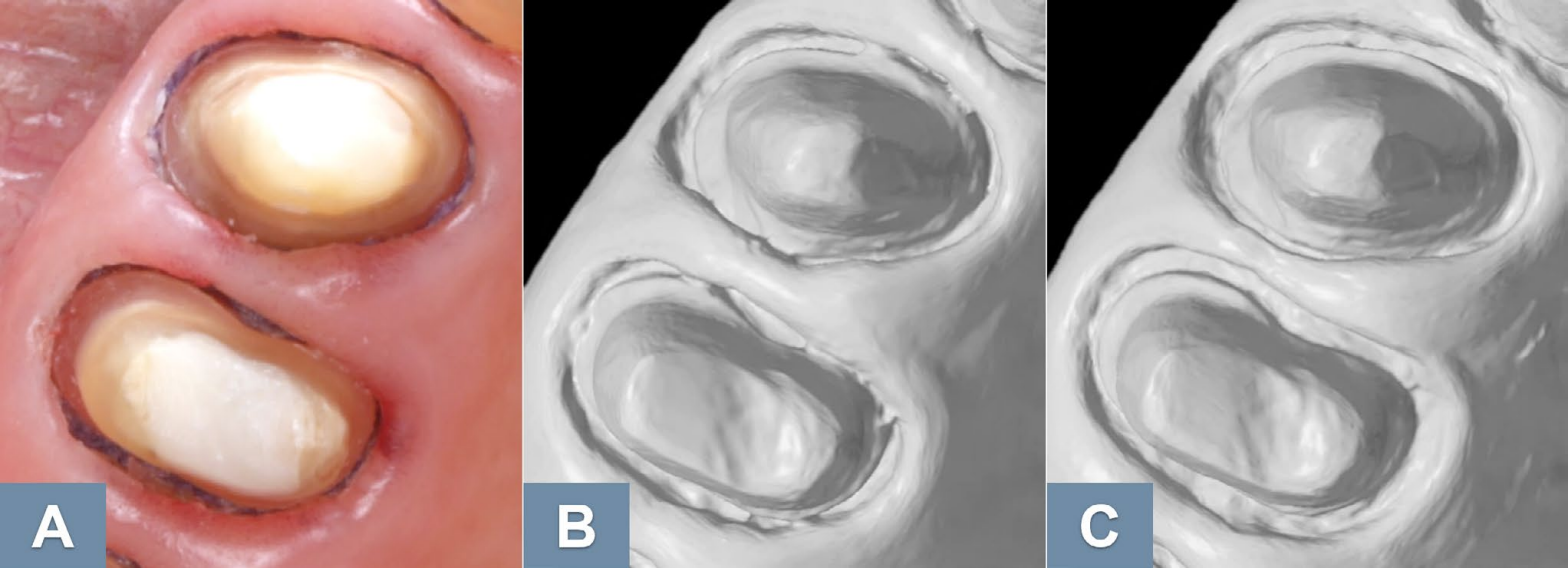
(A) Retraction cord placed prior to intraoral scanning. (B) Bridging artifact [56] obscuring the preparation margins. (C) Clearly visible preparation margins in a second digital impression after proper gingival retraction.

**FIGURE 8:**
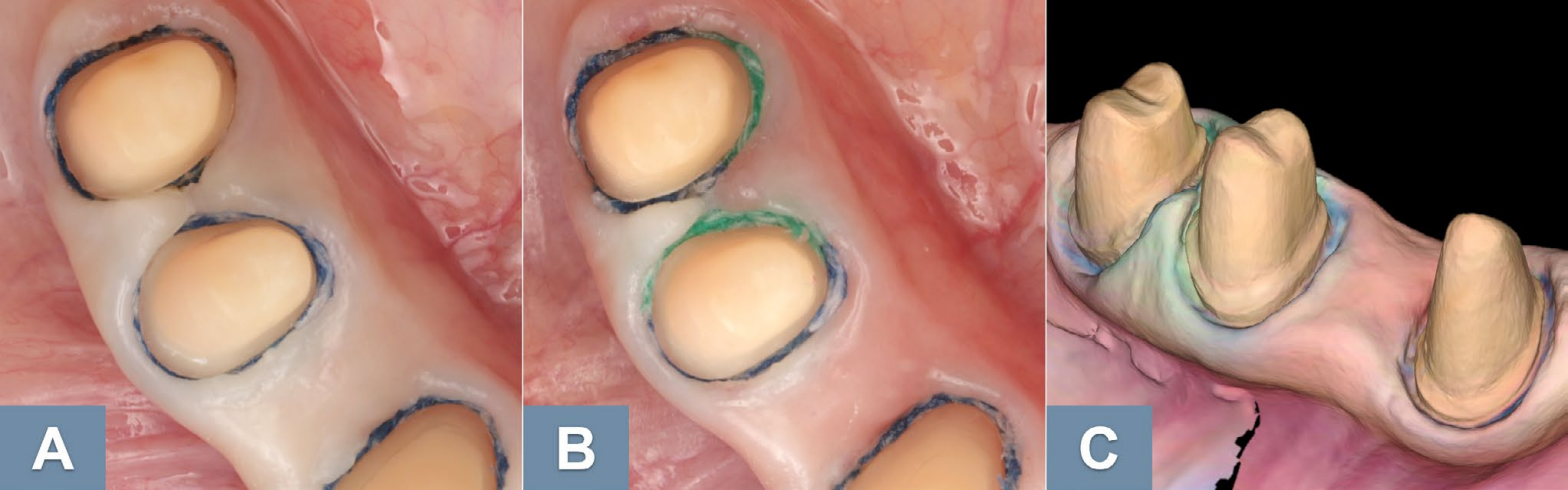
(A) Inadequate gingival retraction. (B) Improved retraction using thicker retraction cords. (C) A digital impression with clearly visible preparation margins.

**FIGURE 9:**
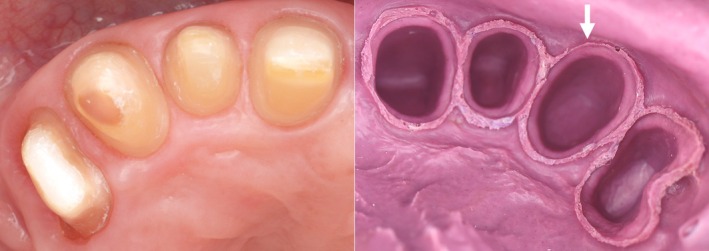
Adequate polyether impression in a region with narrow interdental spaces. The arrow indicates a sulcus area narrowed due to insufficient gingival retraction, yet the impression still captured sufficient detail for clinical use.

Dentists should be mindful that intraoral scanners can only accurately capture structures that are both clearly visible and dry [56]. As such, preparation margins should not be placed too close to approximal teeth [56, 61, 69], since current intraoral scanners struggle to accurately scan deep gaps smaller than 0.45 mm [54, 69]. For the same reason, proper gingival retraction is essential while scanning a tooth preparation as intraoral scanners tend to produce less accurate results when capturing deep subgingival margins [70, 71]. In such cases, a scanning error may occur [56], causing the gingival margin to appear connected to the tooth structure (Figure 7) [56, 69]. In contrast, conventional impression materials can be dispensed while the retraction cords are being removed, taking advantage of the brief window before the gingival tissues rebound, an advantage that digital impressions lack. This suggests that more aggressive gingival retraction may be necessary for digital impressions (Figure 8) compared to conventional methods, which can effectively capture gingival sulcus gaps larger than 0.15 mm (Figure 9) [72]. However, aggressive gingival retraction may lead to gingival recession. As an alternative in certain cases, the deep margin elevation technique [73] may be utilised to avoid scanning deep subgingival margins, thereby reducing the need for excessive retraction.

**FIGURE 10:**
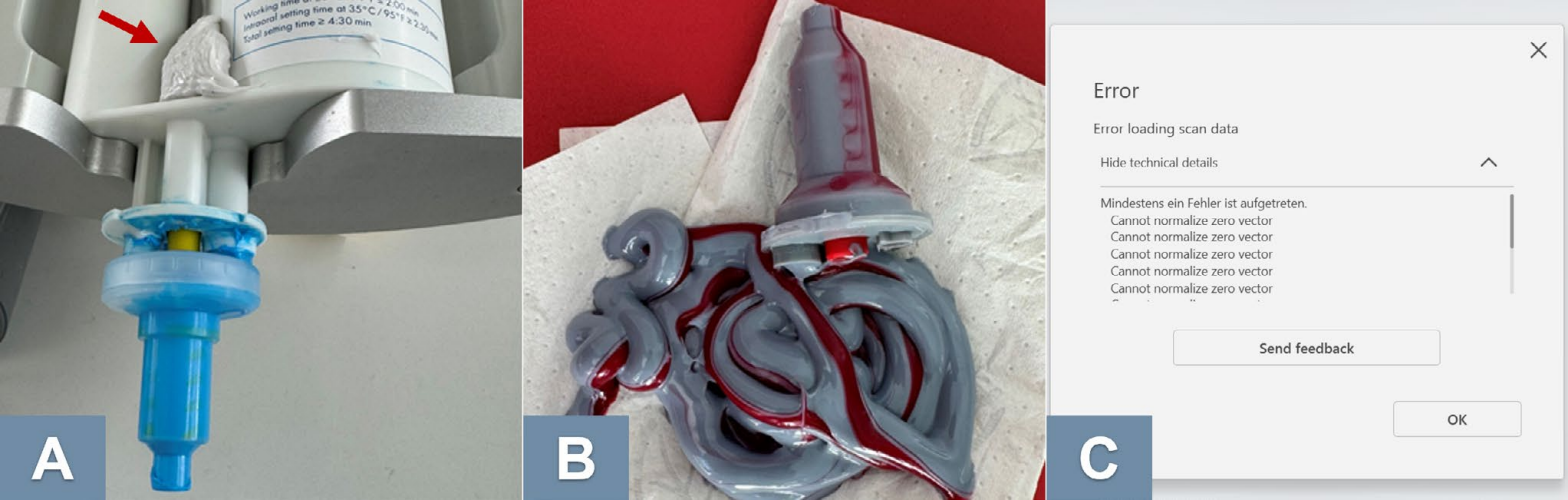
(A) Unnoticed clogging led to improper mixing of the impression material, preventing it from setting and causing significant patient discomfort. (B) Faulty mixing increases strain on both the dental team and patients, particularly if light‐body impression material has already been dispensed intraorally. (C) Software errors present a new challenge in modern dentistry.

Digital impressions require electronic devices, which are subject to wear and tear and will eventually require maintenance or replacement [1]. Another challenge associated with digital impressions is the reliance on commercial software, which gets frequently updated. These updates often require dental teams to adapt to new features or platforms. This also presents a challenge for dental research, as software updates can impact scan accuracy and outcomes [74], making it necessary to interpret results in the literature with caution. Additionally, many of these software solutions require expensive subscriptions, contributing to the overall cost of adopting digital workflows [1]. Furthermore, software instability can also disrupt daily clinical operations (Figure 10).

## 5 Future Directions

The current performance of digital impressions is promising, encouraging their adoption in everyday practice. Moreover, as with many technological innovations, intraoral scanners are rapidly evolving, with continuous improvements in both features and accuracy [4]. For instance, while earlier scanners required powder to capture tooth structures effectively, modern scanners can now capture detailed images without the need for additional materials [4]. While significant advancements have been made, further improvements are still necessary to reliably capture full‐arch scans, translucent surfaces and movable oral tissues. Additionally, features like tooth shade determination [68] and caries detection [4, 75] need to be enhanced to improve their accuracy.

Given the variation in performance among different intraoral scanners [13, 14, 17, 18, 29, 45, 55, 60, 67, 68, 70, 71], careful consideration is essential when making a purchasing decision. Moreover, dentists should familiarise themselves with the specific features of their chosen intraoral scanner, as manufacturers may suggest different scanning protocols or recommendations. As digital workflows become more integrated into daily practice, cloud‐based services are increasingly being introduced to support data processing, transfer and archiving. This approach not only simplifies the workflow but also offers environmental benefits, as clinics no longer need to invest in expensive, separate computing devices or hard drives.

Artificial intelligence is expected to play an increasingly important role in the future of digital dentistry with potential applications across various dental specialties for tasks such as diagnosis, treatment planning, CAD/CAM workflow, treatment monitoring and forensic analysis [4, 18, 45, 65, 75, 76, 77, 78]. Given the slower pace of advancements in conventional impression techniques, it is reasonable to predict that digital impressions, enhanced by AI, will become increasingly integral in the near future. This could pave the way for fully digital workflows in diagnosis and treatment planning, where digital impressions and other diagnostic procedures are seamlessly integrated into a unified digital system.

## 6 Concluding Remarks

While conventional impressions remain essential for certain procedures, such as functional impressions or relining existing dentures, digital impressions have significantly advanced many aspects of modern dentistry, enhancing diagnosis, patient communication, treatment planning and post‐treatment assessment. Intraoral scanners can be recommended for single or short‐span restorations, though they may encounter challenges, such as with deep subgingival margins. The accuracy of full‐arch scans and the ability to capture difficult surfaces also remain areas in need of further improvement. With continuous innovation and the integration of artificial intelligence, digital impressions have the potential to render conventional methods obsolete, while improving workflow efficiency and enhancing the overall patient experience.

## Ethics Statement

The authors have nothing to report.

## Consent

All patient images are from the clinical work of Ahmad Amro Baradee at the University Clinic of Freiburg University and were published with the patients' informed consent.

## Conflicts of Interest

The authors declare no conflicts of interest.

## Data Availability

Data sharing is not applicable to this narrative review.
